# Bone marrow stroma-induced resistance of chronic lymphocytic leukemia cells to arsenic trioxide involves Mcl-1 upregulation and is overcome by inhibiting the PI3Kδ or PKCβ signaling pathways

**DOI:** 10.18632/oncotarget.6265

**Published:** 2015-11-02

**Authors:** Irene Amigo-Jiménez, Elvira Bailón, Noemí Aguilera-Montilla, María José Terol, José A. García-Marco, Angeles García-Pardo

**Affiliations:** ^1^ Cellular and Molecular Medicine Department, Centro de Investigaciones Biológicas, Consejo Superior de Investigaciones Científicas (CSIC), Madrid, Spain; ^2^ Hematology Department, Hospital Clínico Universitario, Valencia, Spain; ^3^ Molecular Cytogenetics Unit, Hematology Department, Instituto de Investigación Sanitaria Puerta de Hierro-Majadahonda, Madrid, Spain

**Keywords:** CLL, stromal cells, arsenic trioxide, Mcl-1, idelalisib

## Abstract

CLL remains an incurable disease in spite of the many new compounds being studied. Arsenic trioxide (ATO) induces apoptosis in all CLL cell types and could constitute an efficient therapy. To further explore this, we have studied the influence of stromal cells, key components of the CLL microenvironment, on the response of CLL cells to ATO. Bone marrow stromal cells induced CLL cell resistance to 2 μM ATO and led to activation of Lyn, ERK, PI3K and PKC, as well as NF-κB and STAT3. Mcl-1, Bcl-xL, and Bfl-1 were also upregulated after the co-culture. Inhibition experiments indicated that PI3K and PKC were involved in the resistance to ATO induced by stroma. Moreover, idelalisib and sotrastaurin, specific inhibitors for PI3Kδ and PKCβ, respectively, inhibited Akt phosphorylation, NF-κB/STAT3 activation and Mcl-1 upregulation, and rendered cells sensitive to ATO. Mcl-1 was central to the mechanism of resistance to ATO, since: 1) Mcl-1 levels correlated with the CLL cell response to ATO, and 2) blocking Mcl-1 expression or function with specific siRNAs or inhibitors overcame the protecting effect of stroma. We have therefore identified the mechanism involved in the CLL cell resistance to ATO induced by bone marrow stroma and show that idelalisib or sotrastaurin block this mechanism and restore sensibility to ATO. Combination of ATO with these inhibitors may thus constitute an efficient treatment for CLL.

## INTRODUCTION

Chronic lymphocytic leukemia (CLL) is characterized by the accumulation of malignant CD5^+^ B lymphocytes in the peripheral blood and lymphoid tissues [1, 2]. Frontline therapies for CLL have been based in the administration of cytostatic drugs (chlorambucil, fludarabine), which, in many cases, control the disease efficiently and are well tolerated [3]. However, patients carrying certain prognostic markers, such as del17p13 or unmutated IgH_V_, do not respond well to these therapies [3, 4]. CLL treatment has greatly improved with the development of more specific agents, such as monoclonal antibodies (obinutuzumab, anti-CD20), kinase inhibitors (CAL-101/idelalisib, for PI3Kδ; ibrutinib, for Bruton tyrosine kinase; sotrastaurin, for PKCβ), or Bcl-2 inhibitors (ABT-263, ABT-199) [3, 5]. These agents are currently in clinical trials or already approved, due to the promising results in most CLL cases. However, the long-term efficacy of these treatments, particularly in refractory CLL cases, is not known. It is therefore crucial to continue searching for new compounds, which could be useful in the treatment of CLL, especially in the advanced setting.

Arsenic trioxide (ATO) is a successfull treatment in acute promyelocytic leukemia [6] and is being trialed in other malignancies, generally in combined therapies [7, 8]. In the case of CLL, we and others have shown that ATO effectively induces *in vitro* apoptosis in all CLL cases tested, including those with unfavorable prognosis [9, 10]. ATO, alone or in combination with other treatments, could thus be an efficient therapeutic agent for CLL.

It is now well established that the CLL microenvironment activate survival pathways on the malignant cells that favor drug resistance and contribute to disease progression [11, 12]. Targeting these pathways has thus become an important issue when studying the effect of cytotoxic drugs on CLL. For example, CAL-101 was shown to down-regulate the chemokine and B-cell receptor signaling induced by stroma and to sensitize CLL cells towards bendamustine, fludarabine, and dexamethasone [13]. Blocking the heat shock protein 90 inhibited the stroma-induced NF-κB signaling and synergistically enhanced the effect of fludarabine [14]. Likewise, blocking PI3K activity regulated the Akt/FoxO3a/Bim axis and increased the cytotoxic effect of fludarabine and bendamustine on CLL cells cultured on stroma [15].

Whether stromal cells influence the response of CLL cells to ATO has not been carefully studied. We recently showed that matrix metalloproteinase-9, a common component of the CLL microenvironment, contributes to the CLL resistance to ATO and fludarabine by preventing downregulation of anti-apoptotic proteins of the Bcl-2 family [16]. Complete understanding of how stromal cells protect CLL cells from the action of ATO will allow the development of strategies that overcome this protection. In the present report we have studied the survival mechanisms induced by stromal cells, responsible for the CLL resistance to ATO. We have also studied whether the modulation of these mechanisms renders CLL cells sensitive to ATO in the presence of stromal cells.

## RESULTS

### Stromal cells protect CLL cells from the apoptotic effect of ATO

**Figure 1:**
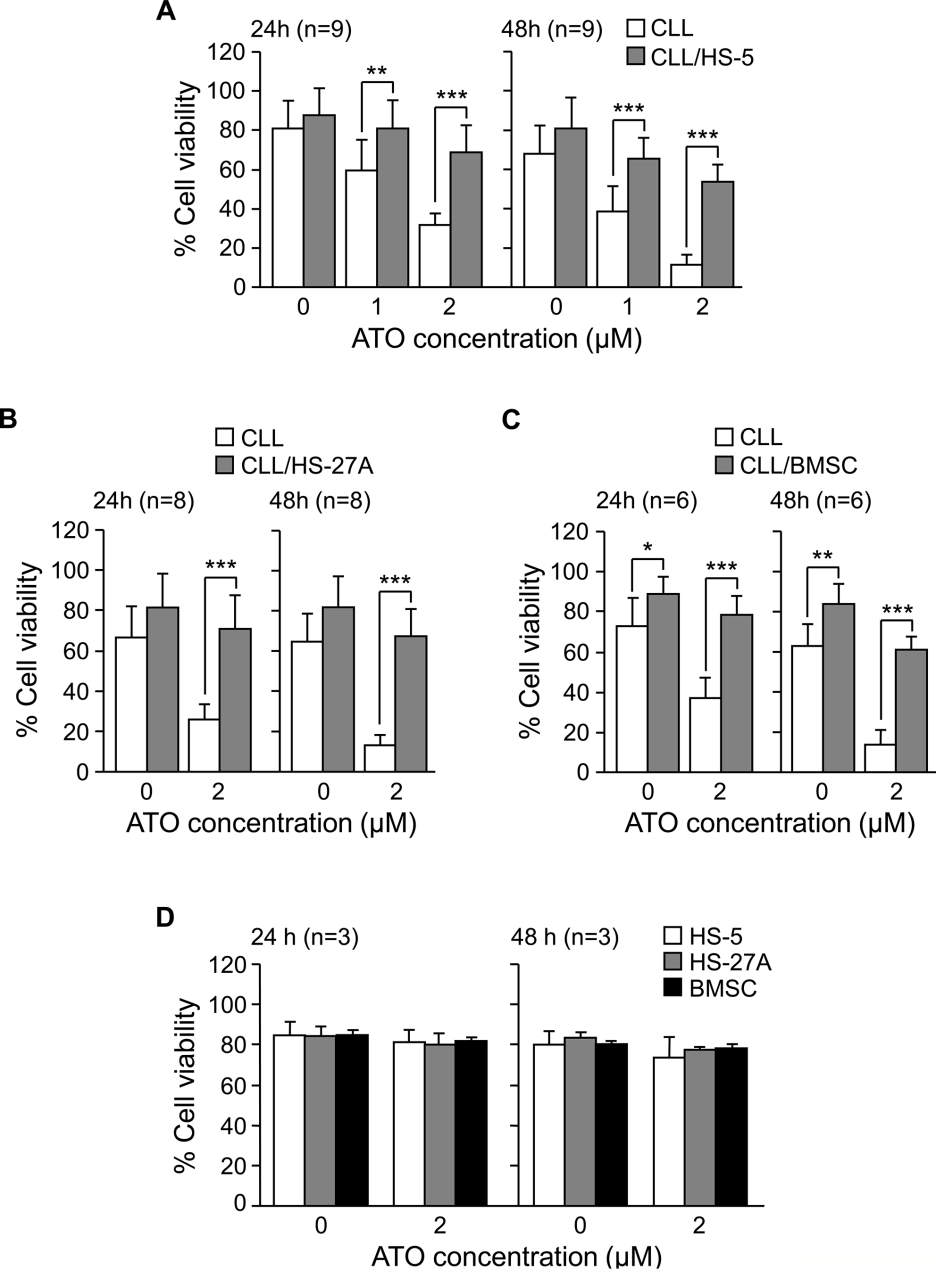
Stromal cells protect CLL cells from the cytotoxic effect of ATO 2 × 10^5^ CLL cells in RPMI/0.1%FBS were cultured in suspension or with HS-5 **(A)**, HS-27A **(B)** or primary BMSC **(C)**. After 2 h at 37°C, ATO was added or not and cells further incubated for the indicated times. Cell viability was determined by flow cytometry, using FITC-Annexin V and PI. **(D)** Confluent HS-5, HS-27A, or primary BMSC were cultured with or without 2 μM ATO for the indicated times. Cells were collected and viability determined as explained. *P ≤ 0.05; **P ≤ 0.01; ***P ≤ 0.001.

To determine if different types of stromal cells influenced the response of CLL cells to ATO, we studied the effect of ATO in co-cultures of CLL-bone marrow stromal cells. In initial experiments, CLL cells from 9 different samples were cultured in suspension or with HS-5 cells (fibroblastoid properties [17, 18]) and treated with 1 or 2 μM ATO. The average constitutive viability of these samples was 82% (range 70–92%) and was normalized to 100. ATO reduced the viability of suspended cells in a dose-dependent manner, resulting in 32% (24 h) and 12% (48 h) viable cells, upon exposure to 2 μM ATO (Figure 1A). No significant decrease in cell viability was observed at earlier times. Co-culture with HS-5 cells significantly protected CLL cells against the cytotoxic effect of ATO. This was already observed using 1 μM ATO and it was clearly obvious with 2 μM, which only reduced CLL cell viability to 69% (24 h) and 54% (48 h) (Figure 1A). All subsequent experiments were therefore performed using 2 μM ATO.

We next study whether HS-27A cells (epithelioid properties [17, 18]) also protected CLL cells from the action of ATO. HS-27A cells prevented CLL apoptosis induced by ATO in the 8 samples studied. Indeed, in the presence of 2 μM ATO, average viability values were 71% (24 h) and 67% (48 h), compared to 26% and 13%, respectively, for suspended cells (Figure 1B).

We also tested the effect of culturing CLL cells on primary stroma derived from a CLL patient. Figure 1C shows that primary stroma also protected CLL cells (6 different patients) from ATO-induced apoptosis, since after 24 h, 2 μM ATO only decreased CLL cell viability to 79%, compared to 37% for suspended cells. After 48 h exposure to 2 μM ATO, these values were 61% and 14%, respectively, for stroma-cultured or suspended CLL cells (Figure 1C). In control experiments, 2 μM ATO was not cytotoxic for HS-5, HS-27A or primary stromal cells, measured after 24 or 48 h (Figure 1D). Altogether these results indicated that stromal cells overcame the apoptotic effect of ATO on CLL cells, thus contributing to their survival and drug resistance.

### The protecting effect against ATO induced by stroma involves interactions with CLL cells through α4β1 and αLβ2 integrins

**Figure 2:**
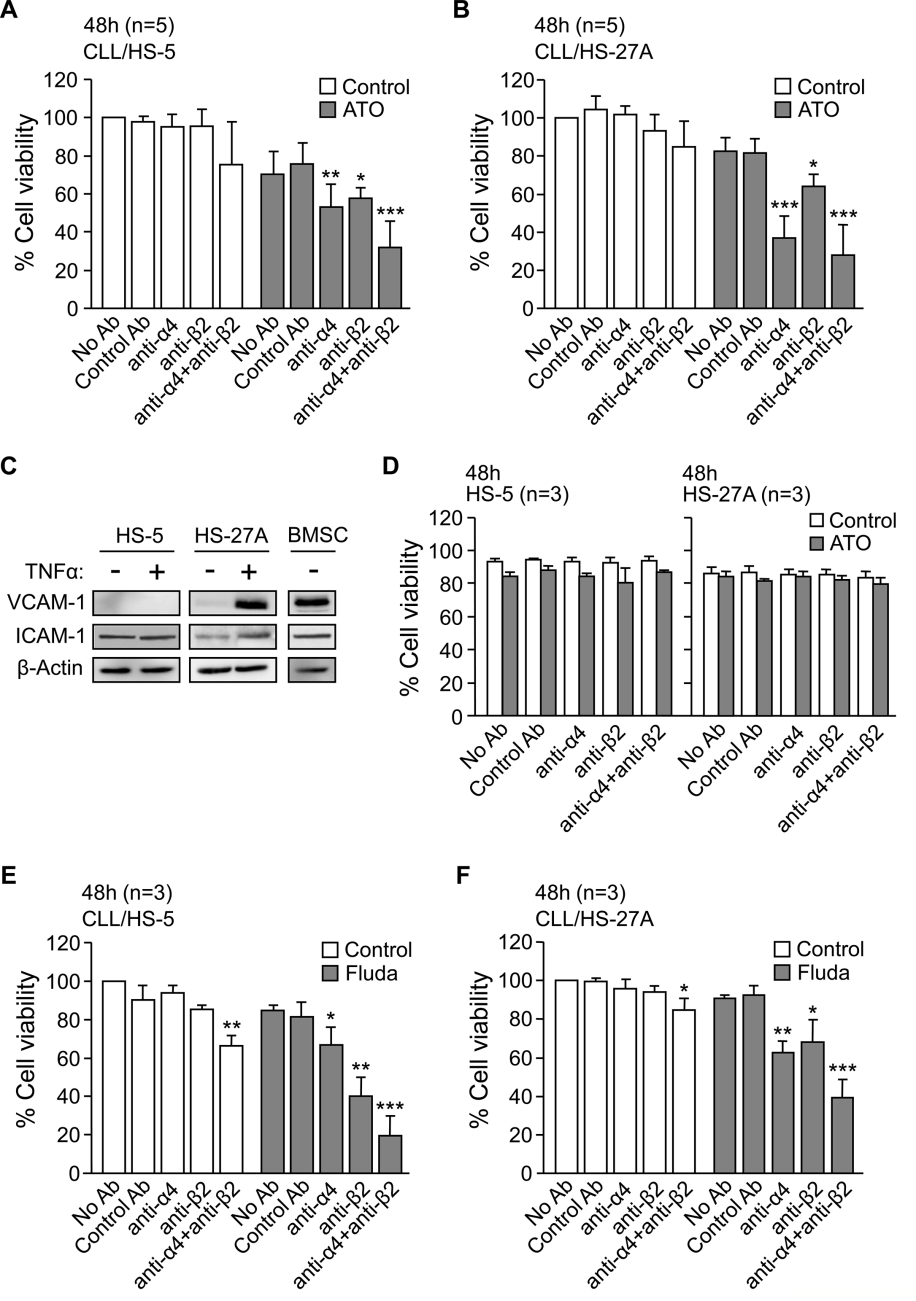
Blocking α4β1 and αLβ2 integrins in CLL-stromal cell co-cultures sensitizes CLL cells to ATO and fludarabine 2 × 10^5^ CLL cells, with or without previous incubation for 1 h with the indicated antibodies, were co-cultured with HS-5 **(A)** or HS-27A **(B)** stromal cells. After 2 h at 37°C, 2 μM ATO was added and viability was determined after 48 h by flow cytometry. **(C)** HS-5, HS-27A, and primary BMSC cells were treated or not with TNFα for 16–20 h and VCAM-1 and ICAM-1 expression analyzed by Western blotting. **(D)** HS-5 and HS-27A stromal cells were incubated for 48 h with the indicated antibodies, in the absence or presence of 2 μM ATO. Cell viability was determined as above. **(E–F)** CLL cells were treated as in (A, B) with the indicated antibodies and added to HS-27A cells. After 2 h at 37°C, 5 μM fludarabine was added and viability determined after 48 h. *P ≤ 0.05; **P ≤ 0.01; ***P ≤ 0.001.

We next studied whether physical contact between CLL and stroma was necessary for the observed survival effect. Because the α4β1 and αLβ2 integrins, present on CLL cells, contribute to these interactions [12], we examined the effect of blocking these integrins on the CLL response to ATO. CLL cells were treated with anti-α4 or anti-β2 blocking antibodies and incubated with HS-5 stromal cells for 48 h, in the absence or presence of ATO. The average viability of control cells (without antibody treatment) at this time was 68% and was normalized to 100. In the absence of ATO, blocking α4β1 or αLβ2 integrins did not significantly reduce the viability of CLL cells co-cultured with HS-5 stromal cells (Figure 2A). However, in the presence of ATO the anti-α4 and anti-β2 integrin antibodies significantly reduced cell viability to 53% and 57%, respectively, while control antibodies had no effect (Figure 2A). The simultaneous blocking of both integrins increased this effect, decreasing the viability of CLL cells to 32% (Figure 2A).

Similar results were obtained for CLL cells cultured on HS-27A stromal cells (Figure 2B). In this case, in the presence of ATO, the anti-α4 antibody was even more effective than in HS-5 co-cultures, reducing cell viability to 37% and to 28% when combined with anti-β2 antibody (Figure 2B). The higher effect of the anti-α4 antibody was probably due to the high levels of VCAM-1 (α4β1 ligand) expressed by HS-27A cells upon stimulation with TNFα ([18] and Figure 2C). In contrast, VCAM-1 was not induced on HS-5 cells (Figure 2C). Additionally, the anti-α4 or β2 integrin antibodies did not reduce the viability of HS-5 or HS-27A stromal cells, either in the absence or presence of ATO (Figure 2D).

We next determined whether the requirement for direct interactions between CLL and stromal cells was a characteristic of the response to ATO or common to other cytotoxic agents, such as fludarabine. In the absence of stroma, 5 μM fludarabine reduced the viability of CLL cells from 72% to 33% (average of 3 samples) after 48 h. At this time, the average viability of control cells co-cultured with stroma was 82% (HS-5 cells) and 84% (HS-27A cells) and was normalized to 100. HS-5 (Figure 2E) and HS-27A (Figure 2F) cells protected CLL cells from the effect of fludarabine, resulting in 85% and 91% viable cells (compared to normalized control), respectively. As in the case of ATO, the anti-α4 or anti-β2 antibodies partially restored the sensitivity of CLL cells to fludarabine in the presence of both types of stromal cells (Figure 2E, 2F). The simultaneous blocking of both integrins was more effective, reducing CLL cell viability to 20% (HS-5 co-cultures, Figure 2E) and to 31% (HS-27A co-cultures, Figure 2F). Therefore, CLL-stromal cell contact via integrins appears to be a general requirement for the drug resistance effect of stroma. Because HS-5 and HS-27A cells behave similarly, subsequent experiments were performed with HS-27A cells.

### Survival pathways induced by co-culturing CLL cells with stromal cells and effect of ATO treatment

**Figure 3:**
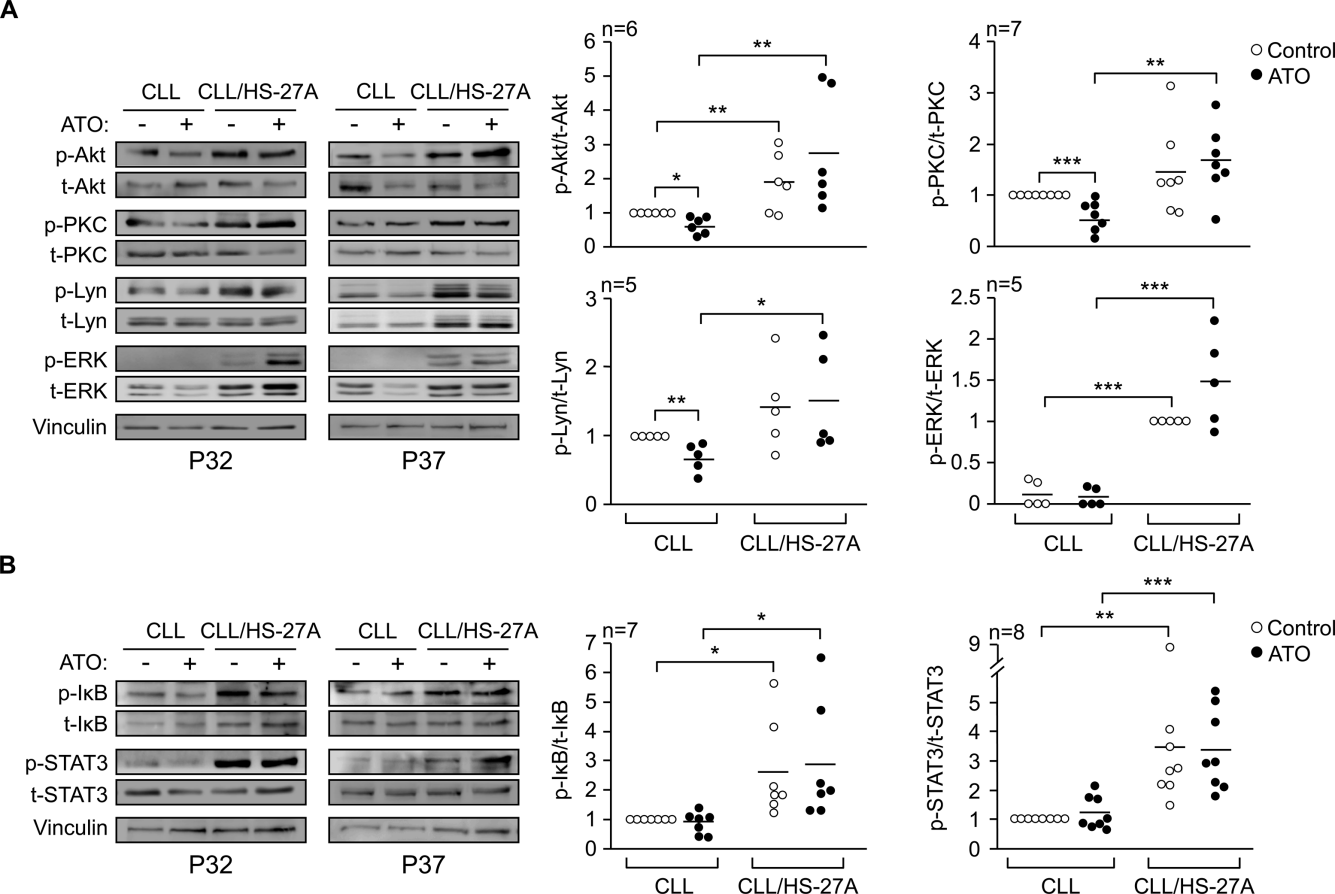
Stromal cells activate several protein kinases and transcription factors in CLL cells exposed to ATO 8–10 × 10^6^ CLL cells in RPMI/0.1% FBS were cultured in suspension or over HS-27A cells for 2 h at 37°C. 2 μM ATO was added or not and cells further incubated for 24 h. Protein kinases **(A)** and transcription factors **(B)** were analyzed by Western blotting. The results from two representative samples and the quantitation of all samples analyzed, after normalizing control values to 1, are shown. Vinculin was also analyzed as a control for protein loading. *P ≤ 0.05; **P ≤ 0.01; ***P ≤ 0.001.

To determine the survival mechanisms contributing to CLL cell resistance to ATO, we first analyzed the possible activation of protein kinases relevant for CLL viability [19]. CLL cells were cultured in suspension or with HS-27A cells for 24 h in the absence or presence of ATO. Western blotting analyses showed that, in cultures of CLL cells alone, ATO significantly reduced the phosphorylation of Akt, PKC, and Lyn, compared to the values of control cells (Figure 3A). In correlation with this, the average viability of these cells decreased from 64% (no ATO) to 28% (ATO treated). Phosphorylated ERK was hardly detectable under these conditions in the samples studied. Co-culturing CLL cells with stroma resulted in the significant activation of Akt, PKC, ERK and Lyn. These kinases remained phosphorylated after 24 h, even in the presence of ATO, indicating their sustained activation by stroma (Figure 3A).

We also examined the activation of NF-κB and STAT3, two transcription factors important for CLL cell survival [20, 21]. NF-κB activation was measured based on the phosphorylation levels of the associated inhibitory protein IκB [22]. The basal phosphorylation of IκB and STAT3 on suspended CLL cells was low and did not change in the presence of ATO (Figure 3B). Phospho-IκB and phospho-STAT3 were significantly increased by stroma and remained elevated upon treatment with 2 μM ATO (Figure 3B). Altogether these results indicated that the survival pathways induced by stroma on CLL cells were not suppressed by treatment with 2 μM ATO.

### Regulation of Bcl-2 family proteins in CLL cells co-cultured with stromal cells and exposed to ATO

**Figure 4:**
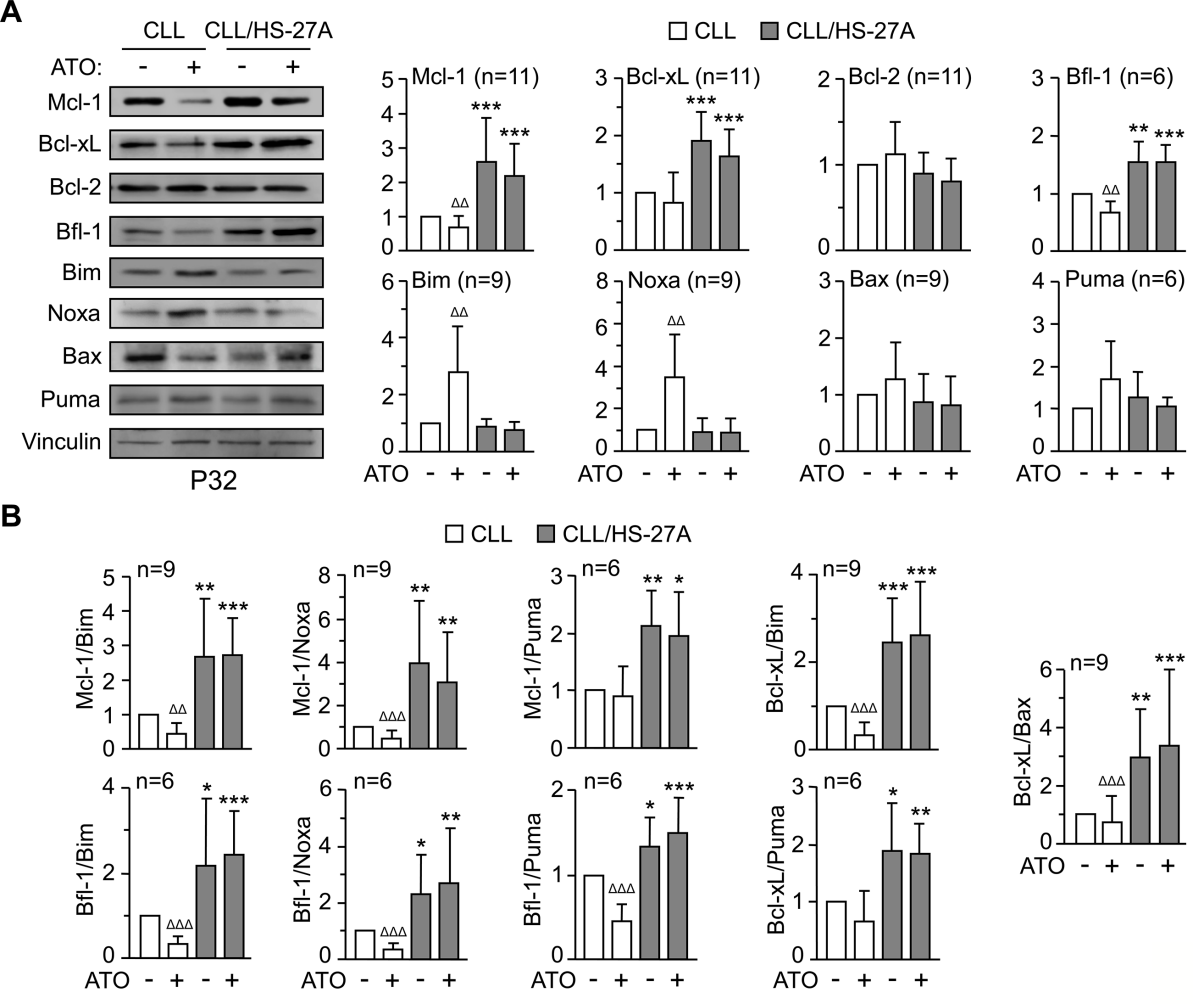
Regulation of Bcl-2 family proteins in CLL-stromal cell co-cultures and effect of ATO **(A)** 8–10 × 10^6^ CLL cells were cultured in suspension or with HS-27A stromal cells. After 2 h at 37°C, 2 μM ATO was added and cells further incubated for 24 h. Protein expression was analyzed by Western blotting. Vinculin was used as an internal control. The results from one representative sample and the quantitation of all samples analyzed, after normalizing control values to 1, are shown. **(B)** Ratios of relevant anti-apoptotic/pro-apoptotic partners of the Bcl-2 family. * CLL cells in suspension vs CLL cells on stroma; ^Δ^ ATO-treated cells vs their respective untreated controls. *or^Δ^ *P* ≤ 0.05; **or ^ΔΔ^ *P* ≤ 0.01; ***or ^ΔΔΔ^ *P* ≤ 0.001.

To further characterize the mechanisms of resistance to ATO induced by stroma, we studied the regulation of Bcl-2 family proteins [23]. CLL cells were cultured alone or with HS-27A cells and treated or not with 2 μM ATO for 24 h. Figure 4 shows the Western blotting results of a representative patient and the quantitation of all patients studied. In suspended CLL cells, ATO significantly diminished the expression of Mcl-1 and Bfl-1 (anti-apoptotic) and upregulated Bim and Noxa (pro-apoptotic), compared to control cells (Figure 4A). This correlated with the lower viability after 24 h of cells treated with ATO (34%) compared to control cells (65%).

Co-culturing CLL cells with HS-27A cells, resulted in a significant increase of Mcl-1, Bcl-xL and Bfl-1, and these proteins remained elevated in the presence of 2 μM ATO (Figure 4A). Stromal cells also prevented the upregulation of Bim and Noxa by ATO (Figure 4A), in correlation with the elevated viability (average 56%) observed on these cells. Moreover, the ratios of anti-apoptotic/pro-apoptotic well-known partners [23] were also significantly increased by stroma and remained elevated upon ATO treatment (Figure 4B). Altogether these results suggested that Mcl-1, Bcl-xL, and Bfl-1 were possible contributors to the resistance of CLL cells to ATO induced by stroma.

### Involvement of the PI3K and PKC signaling pathways in the stroma-induced resistance of CLL cells to ATO

**Figure 5:**
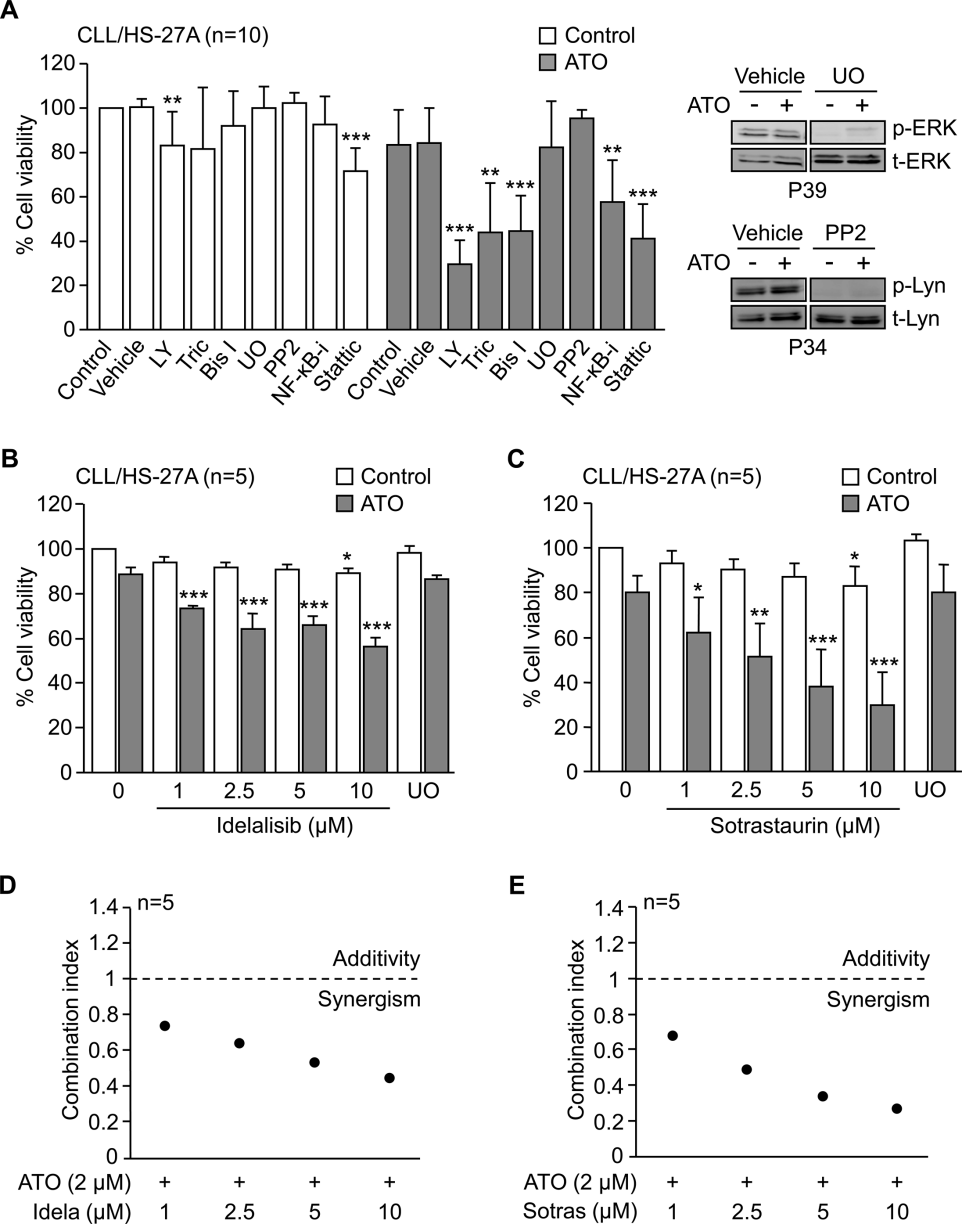
PI3Kδ and PKCβ activities are involved in the stroma-induced resistance of CLL cells to ATO **(A)** 2 × 10^5^ CLL cells were incubated for 1 h with or without the indicated inhibitors and cultured with HS-27A stromal cells. After 2 h at 37°C, 2 μM ATO was added or not, and cells further incubated for 24 h. Cell viability was determined by flow cytometry. LY, LY294002; Tric, Triciribine; Bis I, Bisindolylmaleimide I; UO, UO126; NF-κB-i, NF-κB inhibitor. The inhibitory effect of UO126 and PP2 was confirmed by Western blotting. **(B, C)** 2 × 10^5^ CLL cells were treated for 1 h with the indicated concentrations of idelalisib (B) or sotrastaurin (C) and cultured on HS-27A cells, in the absence or presence of 2 μM ATO. UO was used as control in these experiments. After 24 h, cell viability was determined by flow cytometry. **(D, E)** Combination index values for the interaction of 2 μM ATO with various concentrations of idelalisib (Idela) (D) or sotrastaurin (Sotras) (E) were calculated using the CompuSyn software. Graphs represent the means of five different experiments, each with a different CLL sample. *P ≤ 0.05; **P ≤ 0.01; ***P ≤ 0.001.

To determine which of the survival pathways described above was responsible for the resistance of CLL cells to ATO, we first blocked protein kinases and transcription factors with specific inhibitors. Except for Stattic, that was used at 2.5 μM, the inhibitors were used at 5 μM. This concentration had little effect on basal cell viability (see below) but it was sufficient to inhibit kinase phosphorylation [10, 21]. CLL cells were incubated with inhibitors or vehicle for 1 h prior to culturing with HS-27A cells for 24 h, in the absence or presence of 2 μM ATO. The viability of untreated CLL cells cultured with stromal cells for 24 h (40%–75%) was normalized to 100. Figure 5A shows that in the absence of ATO, only the inhibition of PI3K with LY294002 or STAT3 with Stattic had a limited effect, reducing cell viability to 83% and 72%, respectively. However, in the presence of ATO, blocking PI3K, Akt, PKC, NF-κB or STAT3 activities significantly overcame the survival effect induced by stromal cells (Figure 5A). In contrast, inhibiting MEK/ERK with UO126 or Lyn with PP2 had no effect, albeit these inhibitors efficiently blocked the phosphorylation of these kinases (Figure 5A).

The above results established that PI3K and PKC signaling were essential for the resistance to ATO induced by stroma. Because PI3Kδ and PKCβ are major isoforms in CLL cells and current targets in clinical trials [3, 5, 24, 25], we tested the effect of specifically blocking these isoforms in our system. CLL cells were treated or not with various concentrations of idelalisib or sotrastaurin and cultured with HS-27A cells for 24 h, with or without 2 μM ATO. Figure 5B, 5C shows that, in the absence of ATO, these inhibitors decreased less than 20% the viability of CLL cells cultured on stromal cells. However, in the presence of ATO, idelalisib and sotrastaurin significantly reduced CLL cell viability in a dose-dependent manner, while UO126 behave as the control with no inhibitors (Figure 5B, 5C). The calculated combination index in these assays was < 1 for all concentrations of idelalisib and sotrastaurin tested (Figure 5D, 5E), indicating a synergistic cooperation between ATO and these inhibitors. Blocking PI3Kδ and PKCβ activities simultaneously did not significantly increase the effect of each individual inhibitor (not shown).

### Blocking the PI3Kδ or PKCβ signaling pathways prevents Mcl-1 upregulation and the resistance to ATO induced by stromal cells

**Figure 6:**
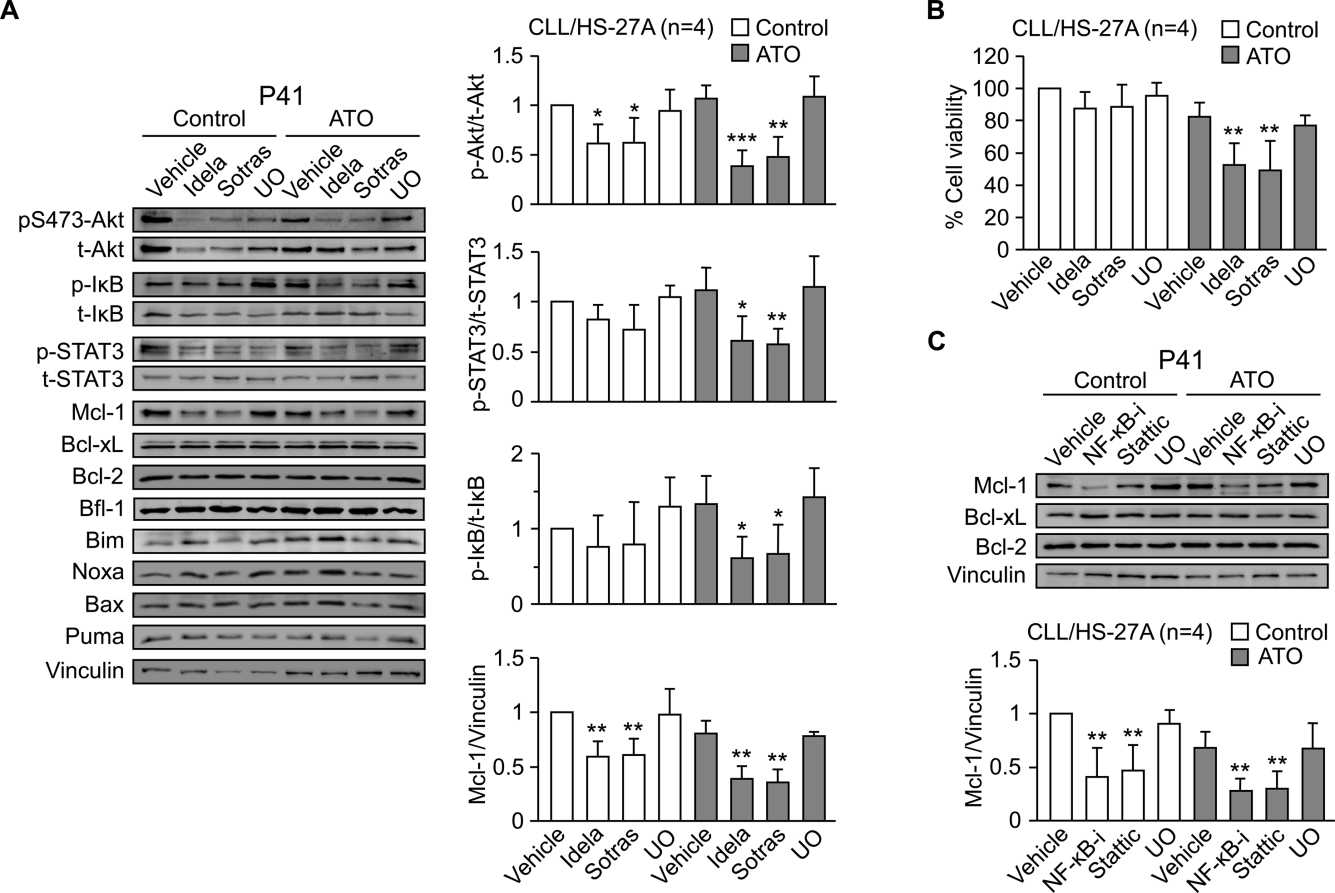
Blocking the PI3Kδ/PKCβ signaling pathways downregulates Mcl-1 and sensitizes stroma-cultured CLL cells to ATO **(A)** 8–10 × 10^6^ CLL cells were treated for 1 h with 2.5 μM idelalisib (Idela), 2.5 μM sotrastaurin (Sotras), 5 μM UO126 (UO), or vehicle, and cultured on HS-27A cells for 24 h, with or without 2 μM ATO. Western blotting analyses of the indicated proteins are shown for a representative patient. The quantitation of proteins with significant changes in their expression on the four samples analyzed (P40, P41, P42, P48) is also shown. **(B)** Average cell viability of the four samples used in (A), determined by flow cytometry. **(C)** CLL cells from the same patients used in (A) were treated or not with inhibitors of NF-κB (NF-κB-i), STAT3 (Stattic), or MEK (UO126) and cultured on HS-27A cells as above. The indicated proteins were analyzed by Western blotting. The results from a representative sample and the quantitation of all samples analyzed are shown. *P ≤ 0.05; **P ≤ 0.01; ***P ≤ 0.001.

To confirm the involvement of PI3Kδ and PKCβ in the resistance to ATO, we first studied whether idelalisib and sotrastaurin inhibited the activation of Akt, NF-κB and STAT3 (see Figure 5A). Both inhibitors were used at 2.5 μM and activation of Akt was determined by the phosphorylation of the S473 residue, a target of PI3K and PKC [26, 27]. The viability of control cells (no inhibitors or ATO) in these experiments was 60% and was normalized to 100. In the presence of ATO, idelalisib and sotrastaurin significantly reduced the stroma-induced phosphorylation of S473-Akt, IκB and STAT3 (Figure 6A). The decrease in S473-Akt phosphorylation was also observed on control CLL cells unexposed to ATO (Figure 6A). However, only in the presence of this agent the dephosphorylation of S473-Akt, STAT3 and IκB correlated with a significant reduction in cell viability to 52% (idelalisib) and 49% (sotrastaurin) (Figure 6B). Blocking MEK with UO126 had no effect (Figure 6A), confirming the specific involvement of PI3Kδ and PKCβ in the mechanism of resistance to ATO.

Further analysis of the same samples demonstrated that idelalisib and sotrastaurin significantly downregulated Mcl-1, both in untreated and ATO-treated cells, while UO126 did not (Figure 6A). As above, only in the presence of ATO, downregulation of Mcl-1 correlated with a decrease in cell viability (Figure 6B). Moreover, idelalisib and sotrastaurin did not significantly reduce the expression of Bcl-xL, Bcl-2 or Bfl-1 (Figure 6A), suggesting a role for Mcl-1 in the resistance to ATO induced by stroma. The pro-apoptotic proteins Bim, Noxa, Bax and Puma did not change under these conditions (Figure 6A).

To then determine whether PI3Kδ and PKCβ regulated Mcl-1 via NF-κB and/or STAT3, we examined the effect of inhibiting these transcription factors. Blocking NF-κB or STAT3 resulted in a significant decrease of Mcl-1, in untreated and ATO-treated cells, while Bcl-xL and Bcl-2 remained unchanged (Figure 6C). Again, the decrease of Mcl-1 correlated with reduced cell viability (55% and 48%, respectively for NF-κB and STAT3 inhibition) only in cells exposed to ATO. In contrast, downregulation of Mcl-1 did not decreased cell viability on control cells (89.5% and 80.2%, respectively, for NF-κB and STAT3 inhibition).

### Key role for Mcl-1 in the resistance of CLL cells to ATO induced by stromal cells

**Figure 7:**
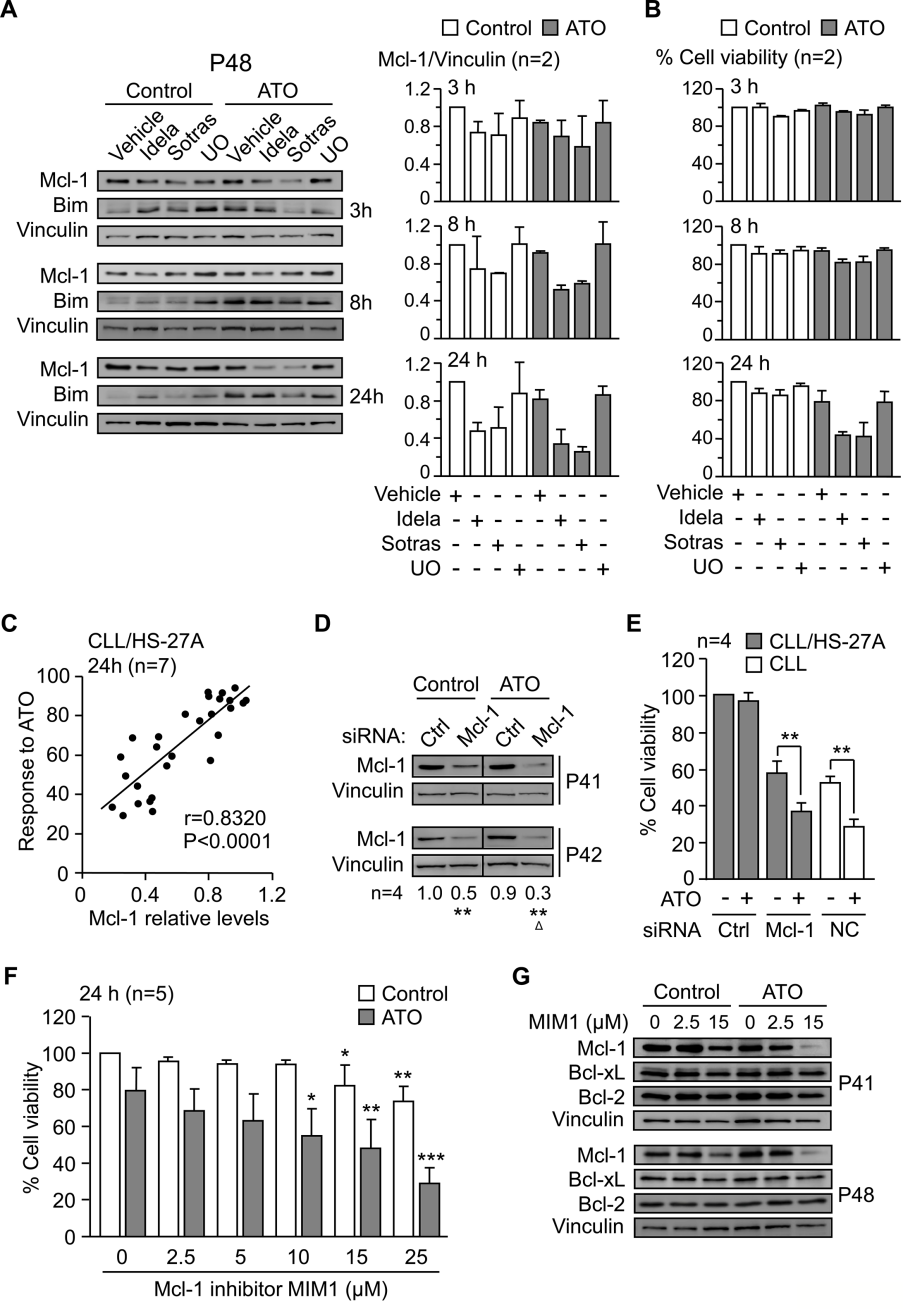
Mcl-1 plays a central role in the mechanism of CLL cell resistance to ATO induced by stroma **(A)** 8–10 × 10^6^ CLL cells were treated with kinase inhibitors as in Figure 6A and cultured on HS-27A cells, with or without 2 μM ATO, for the indicated times. Western blotting analyses are shown for a representative patient and Mcl-1 values quantitated for the two samples analyzed (P41, P48). **(B)** Average cell viability of the same samples used in (A). **(C)** CLL cells (P10, P32, P35, P40, P41, P42, P48) were treated for 1 h with vehicle, 5 μM UO, idelalisib or sotrastaurin (2.5 and 25 μM each), and cultured on HS-27A cells with 2 μM ATO. Cell viability and Mcl-1 expression were determined after 24 h. Pearson's correlation (r) and *P* values are shown. **(D, E)** 24 × 10^6^ CLL cells (P32, P40, P41, P42) were nucleofected with the indicated siRNAs and cultured in suspension or with HS-27A cells. After 16 h at 37°C, 2 μM ATO was added and cells further incubated for 24 h. Nucleofected samples were analyzed by Western blotting (D) and cell viability determined by flow cytometry (E). NC (nucleofection control): CLL cells nucleofected with control siRNA and cultured in suspension. **(F)** CLL cells (P32, P41, P42, P43, P48) were treated for 1 h with or without the indicated concentrations of MIM1 and cultured with HS-27A cells in the absence or presence of 2 μM ATO. After 24 h, cell viability was determined by flow cytometry. **(G)** 8–10 × 10^6^ CLL cells were treated with the indicated concentrations of MIM1 and cultured as in (F). Protein expression was analyzed by Western blotting. *or ^Δ^ *P* ≤ 0.05; **or ^ΔΔ^ *P* ≤ 0.01; ***or ^ΔΔΔ^ *P* ≤ 0.001.

The above results strongly suggested that Mcl-1 downregulation was due to inhibition of the PI3Kδ/PKCβ-NF-κB/STAT3 signaling pathways, rather than a mere consequence of cell death. To confirm this, we measured Mcl-1 levels at shorter times of cell exposure to idelalisib or sotrastaurin, in the absence or presence of ATO. After 3 h of treatment, Mcl-1 expression started to decrease and was reduced 1.8-fold after 8 h in ATO-treated samples (Figure 7A). Cell viability at these times remained unchanged (Figure 7B). After 24 h of treatment, Mcl-1 decreased both in control cells and in ATO-treated cells (Figure 7A). However, only the viability of cells exposed to ATO was significantly reduced at this time, in correlation with the decrease Mcl-1 levels (Figure 7B). Bim expression was also analyzed at these times but no significant changes were observed (Figure 7A). These results strongly suggested that downregulation of Mcl-1 preceded the onset of apoptosis induced by ATO.

We next studied whether there was a direct correlation between Mcl-1 expression and cell viability. CLL cells were treated or not with various concentrations of idelalisib or sotrastaurin and cultured with stromal cells, with or without ATO. UO126 was added as a control in these experiments. Analyses of cell viability and Mcl-1 levels after 24 h demonstrated a strong positive correlation (*P* < 0.0001, *r* = 0.8320) between both parameters (Figure 7C).

To further establish the crucial role of Mcl-1 in the stroma-induced CLL cell resistance to ATO, we blocked the expression or function of this molecule with specific siRNAs or inhibitors. CLL cells (4 different samples) were nucleofected with Mcl-1 or control siRNAs and co-cultured with HS-27A cells for 24 h, with or without ATO. In suspended CLL cells, gene silencing Mcl-1 decreased cell viability to < 10% after 24 h, confirming the importance of Mcl-1 in preventing spontaneous apoptosis of these cells. Western blotting analyses confirmed that the Mcl-1 siRNA significantly reduced Mcl-1 expression in untreated (50% average reduction) or ATO-treated (70% average reduction) cells, compared to control values normalized to 1 (Figure 7D). Because of the stringency of the nucleofection procedure, the viability of CLL cells transfected with control siRNA was 36% after the 24 h co-culture, and this was normalized to 100. Treatment of these cells with 2 μM ATO did not reduce this viability, confirming the protection of stromal cells against this agent (Figure 7E). In contrast, transfection with Mcl-1 siRNA diminished the viability of CLL cells unexposed to ATO to 57.1% (compared to normalized control) (Figure 7E), again reflecting the importance of Mcl-1 in basal cell survival. Exposure to ATO further reduced this viability to 36.5% (compared to normalized control) (Figure 7E). To determine if this was due to the net effect of ATO, we used cells transfected with control siRNA and kept in suspension as a control system. ATO reduced the viability of these cells from 52% to 29% (Figure 7E), and this difference (23%) was very similar to that observed for Mcl-1 siRNA-transfected cells cultured on stroma (19%) (Figure 7E). These results confirmed that the lack of Mcl-1 nearly completely restored the sensitivity of CLL cells to ATO in the presence of stroma.

**Figure 8:**
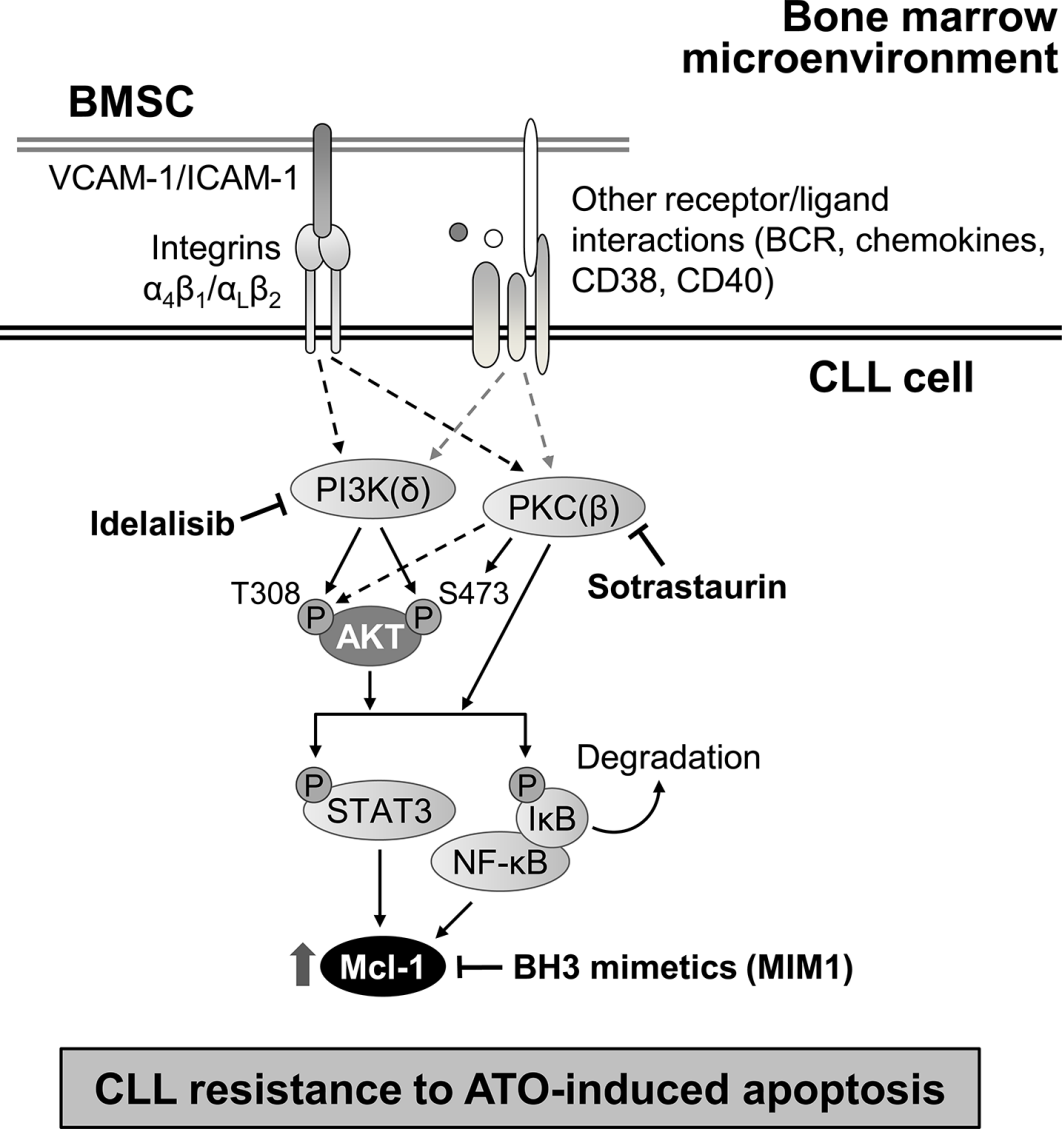
Schematic representation of the identified mechanism of stroma-induced resistance of CLL cells to ATO CLL cells bind to bone marrow stromal cells (BMSC) via several molecular interactions, including those mediated by integrins. The survival pathways induced by stroma involved in the resistance of CLL cells to ATO are depicted. PI3Kδ and PKCβ play major roles in this mechanism and increase Mcl-1 expression. Blocking these kinases with idelalisib and sotrastaurin, or Mcl-1 with specific inhibitors, may be useful strategies to overcome this resistance and render CLL cells sensitive to ATO.

We also study the effect of blocking Mcl-1 function with the specific inhibitor MIM1. CLL cells were incubated with various concentrations of vehicle or MIM1 prior to their culture with stromal cells and treatment with 2 μM ATO. Figure 7F shows that MIM1 sensitized CLL cells to ATO in a dose-dependent manner, reducing cell viability to 29% at the highest concentration tested. To confirm that this was due to Mcl-1, we analyzed by Western blotting the expression of Mcl-1 on the samples treated with MIM1. Figure 7G shows that MIM1 specifically induced the degradation Mcl-1 but not Bcl-xL or Bcl-2, used as controls. Altogether these results established a central role for Mcl-1 in the mechanism of resistance to ATO induced by stromal cells. Figure 8 shows a schematic representation of this mechanism, including the identified survival signals induced by stroma and how their inhibition will render CLL cells sensitive to ATO.

## DISCUSSION

We previously showed that ATO could constitute an efficient treatment in CLL, particularly in combined therapies [10, 16, 28]. To further explore the potential clinical use of this agent, we have now studied the influence of bone marrow stromal cells on the response of CLL cells to ATO. We report that: 1) stromal cells induce CLL cell resistance to ATO via activation of the PI3Kδ-PKCβ/STAT3-NF-κB pathways; 2) Mcl-1 plays a central role in the mechanism of resistance to ATO; 3) blocking PI3Kδ and PKCβ with specific inhibitors overcomes the survival effect of stroma on CLL cells exposed to ATO.

The three types of stromal cells used in study, whether fibroblastoid-like, epithelioid-like or derived from a CLL bone marrow sample, protected CLL cells from the apoptotic action of ATO. This protection mostly involved physical cell-cell contact. Among the several receptor-ligand interactions that take place between CLL and stromal cells [12], our results show that blocking α4β1 and αLβ2 integrins was sufficient to overcome the protective effect of stroma. This is agreement with previous studies showing a role for β1 and β2 integrins in the microenvironment-induced survival of CLL cells [29, 30]. Besides the well-known ligands for these integrins (VCAM-1, fibronectin, ICAM-1) present in bone marrow stroma, an additional cell-cell interaction contributing to the survival effect may be provided by MMP-9. MMP-9 is also present in stroma, is a ligand for α4β1 [31], and has a reported protective role against ATO [16].

It is now well established that co-culturing CLL cells with stromal cells results in reciprocal activation of several signaling pathways, which promote cell survival and drug resistance [12, 32]. In our study, we detected activation of the Lyn, ERK, Akt and PKC kinases, as well as of the transcription factors NF-κB and STAT3. We and others have reported a role for Lyn in CLL cell survival [21, 33]. However, our present results show that Lyn (or ERK) was not involved in the mechanism of resistance of CLL cells to ATO induced by stroma. In contrast, inhibition of PI3K (upstream of Akt) or PKC activities overcame the protective effect of stroma and rendered CLL cells sensitive to ATO. PI3K and PKC were previously shown to play important roles in the survival of suspended CLL cells induced by IL-4 or phorbol esters [34]. Likewise, PI3K and PKC also mediated the survival signals induced by BCR, adhesion molecules and chemokines [35, 36]. Additionally, several inhibitors of the PI3K/Akt and PKC pathways have rendered promising results *in vitro* and *in vivo*, and may potentially have therapeutic application in CLL [5, 37, 38]. Our present results reveal new functions for PI3K and PKC in the resistance of CLL cells to ATO, highlighting the role of these kinases in the response to several cytotoxic drugs.

Further insight into the mechanism of resistance to ATO induced by stroma was obtained by using idelalisib and sotrastaurin as inhibitors for the PI3Kδ and PKCβ isoforms, respectively. The study of these isoforms was chosen because of their predominant expression in CLL and their well-established role in CLL cell survival, as effectors of the B-cell receptor. Indeed, both PI3Kδ and PKCβ are either directly associated to the B-cell receptor (PI3Kδ), or activated by Bruton tyrosine kinase (BTK), another B-cell receptor-associated kinase (PKCβ) [24, 25, 39]. Idelalisib has recently entered clinical trials for CLL [3] and sotrastaurin has yielded very promising preclinical results [5]. Notably, PKCβ-dependent activation of NF-κB has been shown to be crucial for induction of CLL survival by stromal cells [40]. Our present results show that blocking PI3Kδ and PKCβ activities was sufficient to inhibit further downstream signaling (Akt, NF-κB, STAT3 activation) and to overcome the survival effect of stroma. PI3Kδ and PKCβ are therefore key survival pathways in the response of CLL cells to ATO in the presence of stromal cells. The fact that we did not observe an additive or synergistic effect when idelalisib and sotrastaurin were used together, suggests that PI3Kδ and PKCβ are independently activated but converge in subsequent signaling. Indeed, both kinases can directly or indirectly phosphorylate Akt at T308 and/or S473 [26, 41], and activate the transcription factors NF-κB and STAT3 [40, 42]. Additionally, since BTK is upstream of the PKCβ signaling pathway, it is possible that ibrutinib, a BTK inhibitor approved for treatment of CLL [12], will also render CLL cells sensitive to ATO in the presence of stroma.

We have further characterized the mechanism of resistance to ATO induced by stroma by studying the regulation of proteins from the Bcl-2 family. In agreement with our previous observations on the protective role of MMP-9 against ATO [16], our results show that ATO did not increase the pro-apoptotic members Bim, Bax, Noxa or Puma in CLL-stromal cell co-cultures. This differs from the previously described upregulation of some of these proteins by ATO in myeloma [43] and ovarian cancer cells [44]. With regard to the anti-apoptotic members of the Bcl-2 family, our study clearly demonstrates a central role for Mcl-1 in the mechanism of resistance to ATO. This is based on the following evidences: First, Mcl-1 was upregulated by stroma and remained high in the presence of ATO. Second, idelalisib and sotrastaurin decreased Mcl-1 expression in CLL-stromal cell co-cultures treated with ATO, in correlation with a reduced CLL cell viability. Third, inhibition of NF-κB and STAT3, known to regulate Mcl-1 [45], also decreased Mcl-1 levels. This confirmed the involvement of these transcription factors in the mechanism of resistance to ATO and the transcriptional regulation of Mcl-1 by stroma. Fourth, gene silencing Mcl-1 or inhibiting its function, overcame the resistance to ATO induced by stroma. Mcl-1 is a key survival molecule in CLL, recently shown to undergo complex regulation in the context of bone marrow stroma [46]. Indeed, upregulation of Mcl-1 by stroma is central to the protective effect against CLL cell spontaneous apoptosis or apoptosis induced by fludarabine [47], fludarabine/bendamustine [48], or Bcl-2-directed compounds [49]. We also recently reported a role for Mcl-1 in the protection against ATO induced by MMP-9 on CLL cells [16]. Our present results establish that Mcl-1 is a main target to overcome the survival effect of stroma and render CLL cells sensitive to ATO.

In summary, we report for the first time that the mechanism by which bone marrow stromal cells induce resistance of CLL cells to ATO consists in activation of the PI3Kδ-PKCβ/NF-κB-STAT3/Mcl-1 pathways. We further show that the specific inhibitors idelalisib (PI3Kδ) or sotrastaurin (PKCβ), as well as the BH3-mimetic MIM1 overcome the stroma-induced resistance to this agent. Given the promising results obtained with idelalisib and sotrastaurin and the effectiveness of low concentrations of ATO in all types of CLL [9, 10], the combination of ATO with these inhibitors (or with BH3-mimetics) may represent an efficient alternative for the clinical treatment of this malignancy.

## MATERIALS AND METHODS

### Patients and cells

Approval was obtained from the CSIC Bioethics Review Board for these studies. All patients signed an informed consent before blood was drawn. B-lymphocytes were purified from the 48 CLL samples listed in Table 1, using Ficoll-Paque^TM^ PLUS (GE Healthcare, Uppsala, Sweden) centrifugation and, if necessary, negative selection with anti-CD3-conjugated Dynabeads (Invitrogen Dynal AS, Oslo, Norway). The resulting B cell population was mostly >90% CD19^+^, determined on a Coulter Epics XL flow cytometer (Beckman Coulter, Fullerton, CA). Primary stromal cells (BMSC) were obtained from a bone marrow sample of a CLL patient after 3 week culture in IMDM (Lonza, Amboise, France)/20% FBS, and maintained for up to 4 weeks in IMDM/15% FBS. The HS-5 stromal cell line, with fibroblastoid properties [17, 18], was obtained from Dr. Atanasio Pandiella (Cancer Research Center, Salamanca, Spain). The HS-27A stromal cell line, with epithelioid properties and functionally different than HS-5 cells [17, 18] was purchased from ATCC (Manassas, VA, USA). Both cell lines were cultured in RPMI/10% FBS.

**Table 1:** Clinical characteristics of the CLL patients

Patient	Sex/Age	Stage	Ig Status	CD38/ZAP70^a^	α4 integrin^a^ (%)	β1 integrin^a^ (%)
1	M/57	C/IV	ND	−/+	ND	ND
2	M/69	C/IV	ND	−/+	37.0	72.7
3	F/72	C/IV	Mutated	−/+	74.9	99.2
4	M/67	B/II	Mutated	−/+	ND	ND
5	M/65	A/I	Mutated	−/−	78.5	92.0
6	M/79	A/I	Unmutated	−/+	95.1	99.2
7	M/ND	B/III	ND	+/+	82.4	95.0
8	M/79	B/II	Unmutated	+/−	47.3	95.7
9	M/77	A/0	Unmutated	+/ND	45.0	ND
10	F/55	B/II	Unmutated	−/ND	89.0	89.4
11	M/44	B/II	Unmutated	−/+	10.5	63.8
12	M/68	A/0	Mutated	−/+	12.5	41.2
13	M/59	C/IV	Unmutated	+/+	29.9	33.2
14	M/85	C/IV	Unmutated	+/−	25.7	47.1
15	F/73	A/II	Mutated	−/−	75.9	47.8
16	ND	ND	ND	ND	39.8	16.8
17	M/ND	ND	ND	ND	37.0	80.3
18	M/80	B/II	Unmutated	−/+	20.0	37.4
19	F/ND	ND	ND	ND	97.9	17.5
20	M/58	A/II	Mutated	−/ND	50.0	99.0
21	M/48	B/I	Unmutated	+/+	30.0	66.1
22	F/54	B/II	Unmutated	+/−	92.5	82.5
23	ND	ND	ND	ND	ND	ND
24	F/82	C/IV	Unmutated	+/ND	99.2	99.5
25	M/73	B/II	Unmutated	+/+	94.1	98.0
26	F/70	C/IV	ND	+/ND	80.7	78.2
27	M/72	C/IV	Unmutated	+/ND	48.9	54.3
28	M/44	B/II	Unmutated	−/+	18.6	35.0
29	M/61	C/IV	Unmutated	+/ND	60.9	70.9
30	F/69	C/IV	Unmutated	+/ND	99.0	97.4
31	M/79	A/I	ND	−/+	94.0	99.9
32	F/38	C/III	Unmutated	+/+	95.3	91.7
33	F/67	B/II	Unmutated	+/−	97.1	99.3
34	F/55	B/II	Mutated	−/+	98.7	97.0
35	F/65	A/I	Unmutated	−/+	19.8	53.5
36	M/65	B/II	Mutated	−/ND	97.1	92.5
37	M/50	A/0	ND	−/+	40.5	32.5
38	F/69	B/II	ND	−/+	63.2	51.4
39	M/74	B/II	Unmutated	+/+	34.9	51.3
40	M/74	B/II	Mutated	−/ND	26.1	40.9
41	M/73	A/0	ND	−/−	58.3	68.9
42	M/80	A/I	Mutated	−/+	36.2	87.4
43	F/63	A/0	Mutated	−/ND	98.8	95.4
44	M/54	A/0	ND	−/+	95.2	97.0
45	M/ND	ND	Mutated	−/−	45.6	60.0
46	F/45	A/I	Unmutated	−/+	54.3	75.6
47	M/46	A/I	Mutated	−/+	78.4	95.8
48	M/75	B/II	Unmutated	−/+	75.7	73.9

^a^The expression of CD38, ZAP-70 and α4β1 integrin has clinical prognostic value [1, 2]. ND, not determined.

### Antibodies and reagents

Rabbit polyclonal antibodies (RpAbs) to PKC (sc-10800), STAT3 (sc-482), Mcl-1 (sc-819), Bcl-xL (sc-634), Bfl-1 (sc-8351), Bax (sc-526), Noxa (sc-52), and mouse monoclonal Abs (mAbs) to Akt (sc-5298), Lyn (sc-7274), IκBα (sc-1643), Bcl-2 (sc-509), Puma (sc-374223), VCAM-1 (vascular cell adhesion molecule-1, sc-13160), and ICAM-1 (intercellular adhesion molecule-1, sc-107) were from Santa Cruz Biotechnology (Santa Cruz, CA). RpAb to Bim (559685) and mAb to β2 integrin (556084) were from BD Pharmingen (Franklin Lakes, NJ). mAbs to vinculin (V9131) and β-actin (A5316) were from Sigma-Aldrich (St. Louis, MO). mAb to CD19 was from Diaclone (Besançon, France). mAbs against CD38 (16BDH), CD3 (T3B), α4 integrin (HP2/1, function blocking), α4 integrin (HP1/7, inactive control, isotype matched for HP2/1 and anti-β2 Abs), and β1 integrin (Alex1/4) were from Dr. F. Sánchez-Madrid (Hospital de la Princesa, Madrid, Spain). RpAbs to phospho-Akt (T308), phospho-Akt (S473), phospho-PKC (pan, βII Ser660), phospho-ERK1/2 (T202/Y204) and phospho-IκBα (S32/36), and mAb to total ERK1/2 were from Cell Signaling Technology Inc. (Beverly, MA). Rabbit mAb to phospho-Lyn (Tyr396) was from Abcam (Cambridge, UK). mAb to phospho-STAT3 (Tyr705) was from BD Biosciences (Erembodegem, Belgium). HRP-labelled Abs to rabbit or mouse immunoglobulins were from Dako (Glostrup, Denmark). FITC-Annexin V was from Immunostep (Salamanca, Spain). Arsenic trioxide (ATO), fludarabine, and propidium iodide (PI) were from Sigma-Aldrich. Kinase inhibitors PP2 (Src), UO126 (MEK), Bisindolylmaleimide I (BisI, PKC), LY294002 (PI3K), and Triciribine/API-2 (Akt), the Mcl-1 inhibitor MIM1 (#444130), and the NF-κB activation inhibitor (#481407) were from Calbiochem (Darmstadt, Germany). CAL-101/idelalisib (PI3Kδ), sotrastaurin (pan-PKC inhibitor with high efficiency for PKCβ), and Stattic (STAT3) inhibitors were from Selleck Chemicals (Houston, TX).

### CLL-stromal cell co-culture

Bone marrow stromal cells were seeded onto gelatin-coated wells of 96- or 6-well plates. After 4–6 h, HS-27A cells were stimulated with 15–20 ng/ml TNFα overnight at 37°C, 5% CO_2_, to induce VCAM-1 expression. CLL cells (2–5 × 10^6^/ml), with or without previous incubation for 1 h with antibodies or inhibitors, were added to the confluent stromal cell monolayers or cultured in suspension for comparison. After 2 h at 37°C, 2 μM ATO was added or not to the cultures and cells further incubated for various times. CLL cells were gently collected with culture medium for further analysis. The integrity of the stromal cell monolayer was confirmed by phase contrast microscopy. The presence of stromal cells on CLL cell preparations, determined by flow cytometry, was < 1%.

### Cell viability analyses

2 × 10^5^ CLL cells, cultured in suspension or stroma and with or without ATO, were suspended in 300 μl binding buffer (Immunostep) containing 1 μl FITC-Annexin V and 1.5 μg/ml PI. Cell viability was determined on a Coulter Epics XL flow cytometer (Beckman Coulter, Fullerton, CA). For cooperativity analyses, CLL cells were incubated with increasing concentrations of idelalisib or sotrastaurin prior to the addition of 2 μM ATO, and cell viability was determined after 24 h. Synergism or additivity between ATO and these inhibitors was determined using the CompuSyn software (BioSoft, Cambridge, UK). This program allows the calculation of the combination index based on the algorithm of Chou and Talalay (50). Combination index values < 1 indicate synergism, whereas values = 1 indicate an additive effect.

### Western blotting

8–10 × 10^6^ CLL cells were lysed (20 min, 4°C) in ice-cold 20 mM Tris-HCl pH 7.5, 137 mM NaCl, 10% glycerol, 1% NP-40, 1 mM NaF, 1 mM Na_3_VO_4_, containing protease/phosphatase inhibitor cocktails (Roche Diagnostics GmbH, Mannheim, Germany) and analyzed by SDS-PAGE and Western blotting on nitrocellulose membranes (Bio-Rad Laboratories, Hercules, CA). To detect multiple proteins on the same membrane, after identification of the first protein, membranes were washed with TBS/0.1% Tween^®^20 for 10 min, followed by 3 × 30 min incubation in 1% glycine pH 2.2, 1% SDS, 0.0005% NP-40, at room temperature. Membranes were washed 1 × 10 min with TBS/Tween, blocked with 5% BSA for 1 h, and re-probed with subsequent primary and secondary Abs. Protein bands were developed using the enhanced chemiluminiscent detection method (GE Healthcare Europe GmbH, Barcelona, Spain) and quantitated using the MultiGauge V3.0 program (Fujifilm Global Lifescience, Düsseldorf, Germany). Protein load was corrected using vinculin as internal standard.

### RNA interference experiments

The following siRNA sequences were from Sigma-Aldrich: Mcl-1: sense 5′-GGACUUUUAUACCUGUUAUdTT-3′; control siRNA: sense 5′-AUUGUAUGCGAUCGCAGACdTT-3′. CLL cells were nucleofected with siRNAs (200–400 nM/10^6^ cells) in 100 μl Human B Cell Nucleofector^TM^ solution (Lonza) using the U-15 program of the Nucleofector device I (Amaxa, Cologne, Germany). Immediately after nucleofection, 12 × 10^6^ CLL cells were seeded onto wells coated with HS-27A cells or kept in suspension. After 16 h at 37°C, 2 μM ATO was added and cells further incubated for 24 h. CLL cell viability was determined by flow cytometry.

### Statistical analyses

Statistical significance of the data was determined using the two-tailed Student's *t*-test. Pearson's correlation was used to assess the strength and direction of association between cell viability in response to ATO and Mcl-1 expression. The Cohen interpretation of correlation coefficient (r) indicates that 0.10 < r < 0.29 represents a weak correlation; 0.30 < r < 0.49 represents a moderate correlation; and coefficients > 0.50 represent a strong correlation. A *p* value of ≤ 0.05 was considered significant. Analyses were performed using the GraphPad InStat v3.06 software (GraphPad Software, San Diego, CA, USA). All values are expressed as means ± standard deviation.
